# Safety considerations for autonomous, modular robotics in aerospace manufacturing

**DOI:** 10.3389/frobt.2022.1024594

**Published:** 2022-11-18

**Authors:** Christoph Walter, Simone Bexten, Torsten Felsch, Myroslav Shysh, Norbert Elkmann

**Affiliations:** Fraunhofer Institute for Factory Operation and Automation IFF, Robotic Systems, Magdeburg, Germany

**Keywords:** human robot collaboration, operator safety, aerospace manufacturing, safety space, artificial intelligence (AI)

## Abstract

Industrial robots are versatile machines that can be used to implement numerous tasks. They have been successful in applications where–after integration and commissioning–a more or less static and repetitive behaviour in conjunction with closed work cells is sufficient. In aerospace manufacturing, robots still struggle to compete against either specialized machines or manual labour. This can be attributed to complex or custom parts and/or small batch sizes. Here, applicability of robots can be improved by enabling collaborative use-cases. When fixed protective fences are not desired due to handling problems of the large parts involved, sensor-based approaches like speed and separation monitoring (SSM) are required. This contribution is about how to construct dynamic volumes of space around a robot as well as around a person in the way that their combination satisfies required separation distance between robot and person. The goal was to minimize said distance by calculating volumes both adaptively and as precisely as possible given the available information. We used a voxel-based method to compute the robot safety space that includes worst-case breaking behaviour. We focused on providing a worst-case representation considering all possible breaking variations. Our approach to generate the person safety space is based on an outlook for 2D camera, AI-based workspace surveillance.

## 1 Introduction

Our goal is to use the SSM method to implement collaborative applications that allow humans and robots to work as spatially close to each other as possible. To achieve this, the necessary safety distances should be determined as precisely as possible, uncertainties should be minimized, and areas should be dynamically adapted to the situation. Many original works around SSM implementations address one of the sub-problems: collision or distance calculations Glogowski et al. (2019), robot control in terms of stopping or avoiding Ubezio et al. (2021), workspace monitoring in terms of detecting and sensing approaching objects or people Ferraguti et al. (2020). A good overview can be found in Miro et al. (2022). We are trying to work towards a feasible approach for co-design of aspects. This mainly concerns the computation of relevant spaces as well as the sensory workspace monitoring with the help of cameras in a form that promotes the interaction of the two aspects. With respect to the control of the robot, we do not rely on any kind of avoidance behavior, which in turn results in more complexity in the interaction with approaching persons and can thus be a source of uncertainty. Instead, we use the approach of stopping the robot as fast as possible. This approach should be implemented by all other behaviors anyway as a fallback possibility (failure of the equipment during the safety-monitored avoidance movement), whereby these systems can basically not be better in terms of improving the cooperation with the human by further reduced distances.

In aerospace manufacturing, the handling of large parts is a common occurrence Caterino et al. (2021). There is also low throughput compared to other industries. This leads to parts being stationary for some time while work is taking place around. A lot of work is carried out by human workers. When introducing robot-based automation for some of the tasks, the capability of close human-robot-collaboration and co-existence is beneficial Costanzo et al. (2022), Meißner et al. (2018). When reducing the overall robot speed is not desired, this leaves the options of minimizing separation distance by eliminating uncertainties, making it dynamic, and using capable sensors (German Commission for Electrical, Electronic and Information Technologies of DIN and VDE (2021)). Sensors should then be able to monitor position and movement of the persons in question in detail. Investigations of separation distances using different approaches were covered in the past Lacevic and Rocco (2010), Vicentini et al. (2014). More recent work also tries to exploit advances in pattern detection and recognition for safety applications Costanzo et al. (2022).

In the following section of this work we present a brief analysis of a particular use-case of intelligent robotics applicable to pre-assembly as well as final assembly of aircraft structures. The use of both fixed as well as mobile robots are being considered. The application is covering the fastening of HI-LOK™ collars. Here, it is beneficial to employ human co-workers in parallel with robots Caterino et al. (2021).

Next, we propose a method for the dynamic generation of first spatial volume around the robot based on pre-planned movement. This is a vital step for implementing a flexible and safe SSM-system. The need for dynamic generation of separation distance is also emphasized by the dynamic behaviour of the robot using autonomously generated actions based on models and environment perception.

In the final part, we discuss how to detect and monitor the presence of persons in the vicinity using optical sensors. We discuss the possibility of using artificial intelligence (AI) based detection of humans using cameras. Furthermore, we present our current approach to construct another spatial volume representing the human based on a projection of the convex hull of the image space silhouette onto the ground floor.

## 2 Analysis of a collaborative application

**FIGURE 1:**
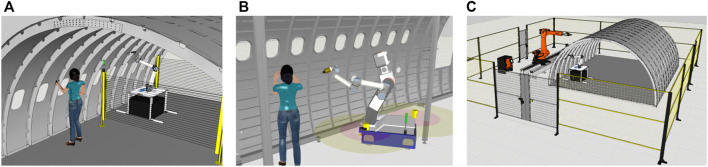
Co-existence with human workers using fixed sensor placement for separation **(A)**; Autonomous robot with fully dynamic safety space **(B)**; Implementation using a non-collaborative setup **(C)**.

When we consider safety of robotics systems, it is mandatory to follow the principles laid out in the Machinery Directive EC (2006). The risk assessment is therefore specific to a particular implementation of a robot system, but contains reoccurring risks and mitigation measures. A major source of risk are mechanical hazards, like the collision of the robot with a person. Speed and separation monitoring aims to mitigate that risk by preventing the robot to contact a person close by while in motion. To better understand safety requirements, and in particular to evaluate implementations and possible improvements of speed and separation monitoring, we considered several possible implementations of the same application. We decided on the fastening of HI-LOK™ collars as the application. We consider this application because it is a common type of fastener used on many different parts of the fuselage. Some are more difficult to reach then others. Therefore it presents a suitable case for combining the different strengths of human workers and automatic solutions for working on the same product and in conjunction with shared work spaces (Figure 1).

We considered three possible variants: A non-collaborative implementation using fences (Figure 1C), a fixed robot with light curtains (Figure 1A), and an autonomous mobile robot with dynamic safety space (Figure 1B). The first variation would employ a large robot or a robot with a workspace extension *via* a linear unit in order to cover large shell pieces. It has no further implications for SSM. The other two variations use a small or medium sized robot which needs to be relocated multiple times in order to cover a large part. The third variation in particular would use a smaller type of robot because the mobile platform can easily move between each fastening step which in turn requires less reach of the actual robot arm. Here, power and force limiting would also be a strategy to mitigate collisions. In our case, SSM is still preferable since it better covers a wider range of tool related hazards, including non-mechanical ones. In this implementation, the robot needs to be made autonomous. It can reposition itself along the whole part as required based on situation dependent decision making. A human worker follows along at a distance in order to cover remaining work (parts not reachable for the robot). This however, requires dynamic repositioning of monitored safety zones. Furthermore, it is beneficial for the separation between robot and co-worker to be as small as possible in order for the worker to finish work shortly after the robot. This also minimizes the risk of inadvertently triggering safety stop by the worker. To summarize, the fully automated version behind fences is cumbersome when it comes to moving the part in and out. The other two versions show, that it is beneficial to reduce the necessary space between worker and robot in order to cut total time required. This is because larger separation distance results in less work to be done in parallel on the same part.

## 3 Robot safety space generation

In our approach we split the separation distance in two parts: “robot safety space” and “person safety space”. Both terms are not to be confused with other terms found in the literature or in robot manuals like “maximum space”, “operating space”, “restricted space”, and so on. Here, robot safety space identification is the task of calculating the volume of space that may be occupied by a moving part of the robot at a certain point in time in case of occurrence of a stop condition. For a typical time triggered system this is aligned with activation times and is true for the duration of the cycle time. The stopping motion can be described by a swept volume. Similar swept volumes have been used for ensuring safety of whole movements in the past Täubig et al. (2011), but are usually used to cover a planned trajectory instead. The volume includes also the tool and parts attached to the tool. The robot safety space depends on the state of the robot, that is to say, on its point in time on the executed trajectory. It also depends on the performance characteristics of the equipment, like reaction times and braking capabilities Marvel and Norcross (2017). The contributing factors are laid out in the technical specification ISO/TS 15066 ISO (2016). For SSM, it provides a formula (Eq. 1) consisting of several summands for calculating the minimum required distance between any human worker and the robot system. It aims to stop the robot before an approaching human can touch the robot. It does not consider evasive movements. Nevertheless, a remark should be made that SSM cannot prevent humans from colliding with a robot that is in a stationary position after stopping. The separation distance $\left(S\left(t_{0}\right)\right)$ is calculated by considering the speed of the approaching human (*v*_*H*_) in conjunction with the complete chain of reaction times (*T*_*R*_) of the equipment plus the time required for stopping the robot (*T*_*S*_). This part of the equation requires knowledge of the position and speed of persons in the working area. However, if the speed is not known, a worst-case speed can be used instead. Uncertainty of the persons position (*Z*_*D*_) as well as an additional margin for sensor resolution (finger, hand, arm) (*C*) are added here as well. Another part of the sum is the distance and speed (*v*_*S*_) as the robot moves towards the person during its stopping motion. Here, information provided by the manufacturer to describe the breaking performance is used. For the maximum robot speed in the direction of an operator in the collaborative workspace (*v*_*R*_), we consider the current speed on its trajectory, which must be known and ensured. Again, some distance representing the uncertainty of the robot position (*Z*_*R*_) is added.

$$\begin{array}{ll} S\left(t_{0}\right) & \geq \left(\int_{\tau =t_{0}}^{\tau =t_{0}+T_{R}+T_{S}}v_{H}\left(\tau \right)d\tau \right)+\left(\int_{\tau =t_{0}}^{\tau =t_{0}+T_{R}}v_{R}\left(\tau \right)d\tau \right)\\ & \;+\left(\int_{\tau =t_{0}+T_{R}}^{\tau =t_{0}+T_{R}+T_{S}}v_{S}\left(\tau \right)d\tau \right)+\left(C+Z_{D}+Z_{R}\right), \end{array}$$ (1)

There are some shortcomings to the standard approach when it comes to a person’s position and speed. If the person was guaranteed to be completely stationary, the robot could move in a way that it would stop directly in front of the person. In practice, this cannot be assumed. The stipulated assumption of 2 m/s for the person speed leads to a considerable distance requirement. This can only be countered by implementing a system with fast reaction times in conjunction with a slowly moving robot in order to minimize the stopping times. But not only faster reaction times of the equipment could bring worker and robot closer together. The simplification of reality that was used for the mentioned distance formula means that every body part of a person is treated the same. In contrast, workers may actually move their limbs, especially the arms, quite rapidly. This results in transient high speeds, exceeding the stipulated 2 m/s while being limited by the reach of the particular limb if the torso is not starting to move in the same direction as well. This leads to exaggerated separation distances. However, this only becomes a problem when actually performing live speed monitoring.

In our approach, we consider the robot and the person safety space separately. The advantage is, that each part can be adapted to the needs or circumstance associated with either the robot or the sensor system used for detecting persons. However, the robot safety space is not completely independent from the sensor used. The sensors response time is also a contributing factor for the safety space. During sensor latency, the robot would move according to its designated trajectory. This means that we have to distinguish between occurrence of a stop event, i.e., the intersection of robot and sensor safety space, a trigger signal between both sub-systems, and the start of a stopping motion.

Knowledge of the robot safety space is important for setting up an SSM-based HRC application. For a static set of pre-programmed trajectories, it is possible to consider the overall worst-case volume whereby all possible behavior variations of the robot are covered when stopping at any time during motion. In this case, safety barriers like light curtains can be placed at design-time to encapsulate the safety space. Although sensor performance, including spatial resolution and latency, need to be considered as well, this is a straight forward process. In the case of a dynamically generated movement, safety space is ideally done at run-time. Another possibility of handling dynamically generated motions would be to design it for a border-case and to perform a run-time check, whether or not the generated motion would be within these limits. The third case is the use of a more complex sensor systems which introduces constraints like occlusion. Here, the combination with dynamically generated motions is also possible. In order to deal with this general case, an online safety space calculation seems the most promising approach. In our case, we propose a voxel-based discretization in conjunction with a breaking model that covers not only a controlled stop, but also handles the case of departing the pre-determined trajectory by using dedicated (friction) breaks. This leads to larger safety spaces than assuming only the ideal breaking situation. By using a precise geometric model as well as the exact trajectory followed by the robot we can minimize the respective terms of the separation distance calculation.

**FIGURE 2:**
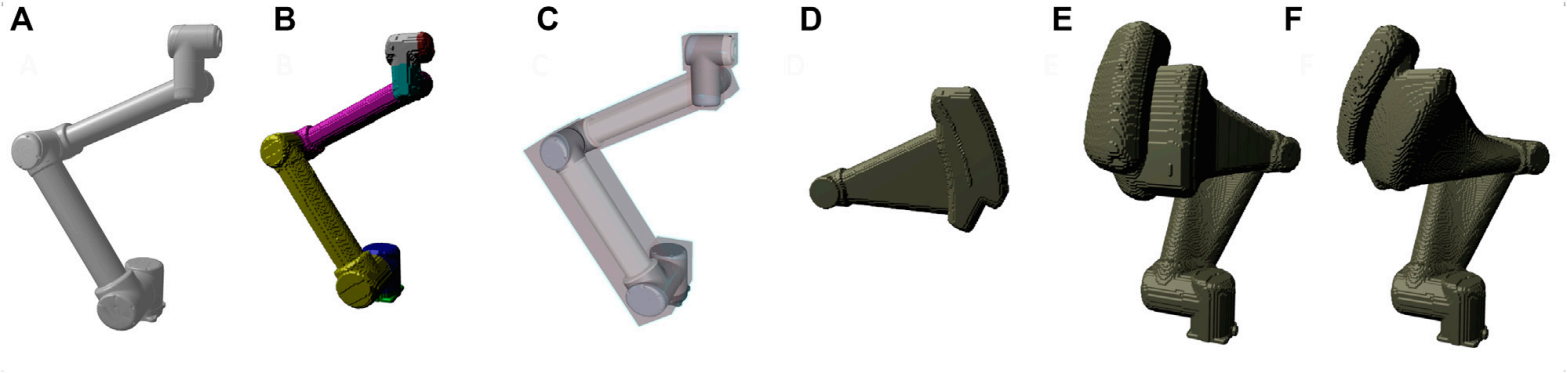
Intermediate steps of the robot safety space generation **(A–F)**; Polygonal representation **(A)**, Voxel Representation **(B)**, Simplification using bounding boxes **(C)**, Swept volume when breaking 3rd joint **(D)**, Combined result of braking 1st and 3rd joint **(E)**, Space covered by ideal controlled stop **(F)**.

We consider two different object types: Environment objects that can be considered as static, and dynamic collision objects (DCOBJ). These are links of serial robots for which the voxelization is done by additionally applying breaking calculations based on the specific robot model as well as its current motion state. It also includes attachments like tools or large parts. To process DCOBJs, we implemented a multi stage approach. It is based on a fast voxelization capability as illustrated in Figure 2. At first, the swept volume representing the part of the trajectory covered during reaction time of the detection system is generated. Here, the links are incrementally moved according to the pre-panned trajectory and the corresponding voxels are marked as occupied. Next, during an iterative process starting from the tool and working its way link by link backwards to the robots base the swept volume of the actuated link is generated and saved to a separate voxel structure. For all consecutive links, the previously generated swept volume is added to the added polygonal model of the currently actuated link. Rasterization of polygonal models as well as resampling of voxel structure from the previous iteration is done by applying conservative rasterization. This prevents thin primitives to partially disappear because they may not cover the voxel center. These steps aim to create a volume structure not only representing the robot geometry at a single point in time on its trajectory, but also the space potentially required when breaking from that exact moment until standstill in all combinations of breaking distances for each link. This gives us the worst-case volume of space that may be occupied by a moving part during breaking. For this, information on the breaking performance of the robot under the specific circumstances is required. The robot type, attached payload, joint configuration, and speed of movement influence the breaking time and the residual movements of each of the individual joints. While breaking at slow speed can be nearly instantaneous, the kinetic energy that needs to be dissipated when breaking at full speed is much higher. This puts stress not only on the motors or breaks and on the overall structure of the robot but also on the mount or fixture where the robot is attached. The many contributing factors lead to typically conservative specifications of worst-case breaking distances by robot manufacturer. Usually, you can find a table within the documentation that provides the necessary information for exemplary payloads, speeds and extension. The extension basically refers to the distance of the payload from the base. For the given starting point on the trajectory we look up the worst-case bracket from the provided table and use the resulting information as input for our swept volume calculation. In a final step, uncertainty of robot position is added to the voxel structure by marking all voxels within that distance to occupied voxels also as occupied. The algorithm is provided in pseudo-code in 1. In case of a sensor guided movement, the planned trajectory gets perturbed during execution by the sensor input. Our approach could handle such applications as well by sampling from all the possibilities of typically 2D sensor input. Even though inefficient, this covers amplification of Cartesian deviations at the tool by robot structure and could be optimized in the future.

Algorithm 1. Robot Safety Space Generation

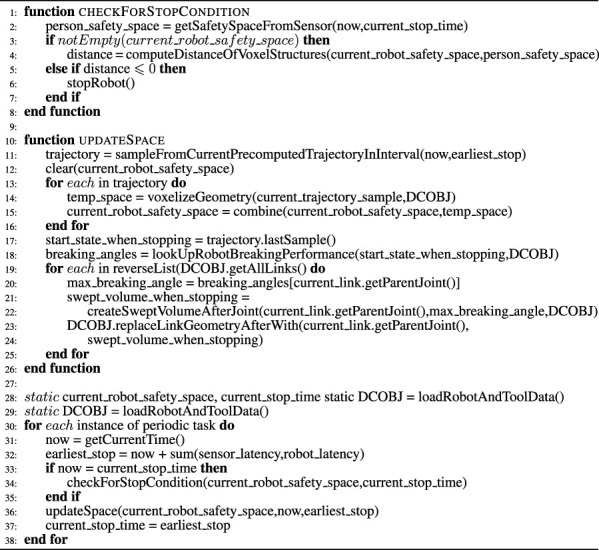

To summarize the robot safety space generation, two points are notable: The use of detailed polygonal models of the robot and attachments is beneficial compared to using coarse approximations in the form of few geometric primitives (see A and C) in 2. That is to say, simplification is implicit when converting the polygonal models to a voxel representation. The advantages are, that no additional collision models are needed, and no unnecessary padding is included in the generated volume. Another notable fact is, that our algorithm easily generates the volume that covers all possible variations of breaking behavior. While the resulting volume is usually larger then the ideal behaviour in stop category 1 or 2 (see E and F in 2), the result provides better safety because it also covers category 0 stops as well.

## 4 Workspace monitoring and person safety space

For generating a detailed representation of the state of the person, we considered the capabilities of a camera based detection. Cameras deliver rich information with high spatial resolution. They can also be made with high frame rates and thus small reaction times, which is of particular importance for the workspace monitoring as we laid out in the previous section. Significant progress has been made when it comes to object detection by applying artificial intelligence (AI) based on machine learning.

The perception of humans in the workspace area of robots is required to rate situations differently. Camera-based systems like 2D cameras (color, gray scale) or depth cameras (RGB-D cameras) are used to capture the robots environment. The evaluation of the data from the camera system can be done with the latest AI-based systems. Here, machine learning (ML) methods such as deep learning are particularly suitable for solving the various tasks in image recognition such as the identification of a variety of objects in cluttered environments or in changing lightning than classical image processing methods.

**FIGURE 3:**
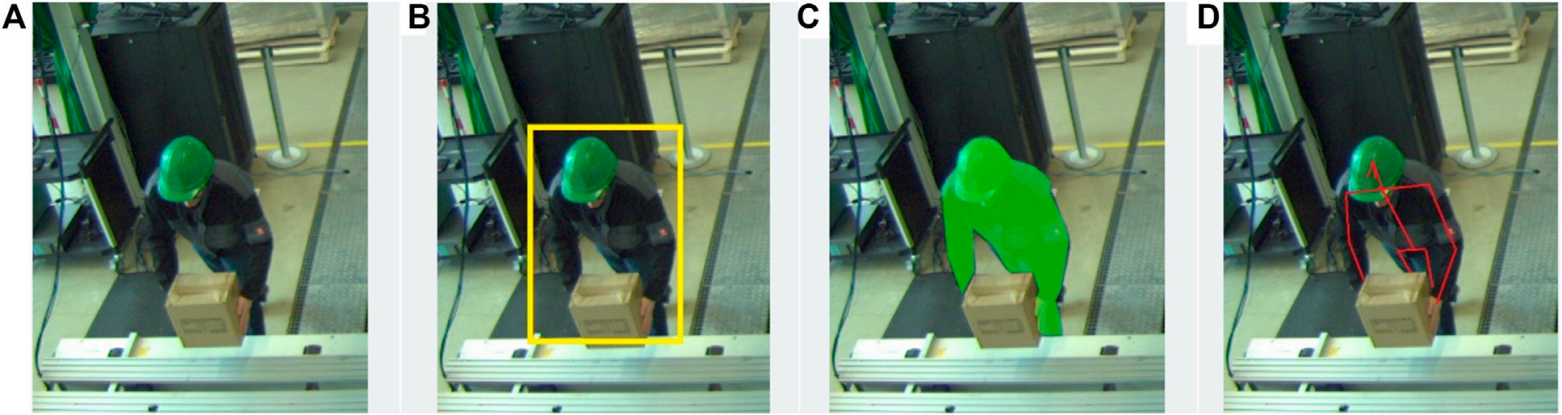
Various computer vision tasks for humans detection: classification**(A)**, detection**(B)**, segmentation**(C)**, skeleton **(D)**

There are a wide range of tasks in computer vision, and to determine which model can solve which tasks, we need to define the tasks what we want to solve (Figure 3). The simplest tasks for camera-based data is image classification (Figure 3A), in which only a single camera image is considered: if a person is recognized here, the system must activate an emergency stop, classification models were introduced by Krizhevsky et al. (2012) Simonyan and Zisserman (2014) or Huang et al. (2017) and the result refers to the whole image. To extract more detailed information from the image data, other methods for detection (Figure 3B) and segmentation (Figure 3C) can be used to analyse the robot environment. The detection networks localize objects in the image with the additional information of the classification within the estimated 2D-pixel coordinates (Ren et al. (2015), Liu et al. (2016), Redmon et al. (2016), Duan et al. (2019), Tan et al. (2020)). In Long et al. (2020), these models were tested on the MS-COCO data set of Lin et al. (2014) with various objects and people. As ML methods and models are constantly evolving, this provides a general overview of the method’s performance. Next, algorithms for pixel-wise classification (segmentation) are used to separate objects from the background. Representatives of segmentation networks were introduced by Ronneberger et al. (2015), He et al. (2017), Badrinarayanan et al. (2017) and Wang et al. (2020). Other models determine the segmentation of the individual body parts such as Lin et al. (2017), Güler et al. (2018) and Oved (2019). Additionally, the recognition of the human’s kinematic state is beneficial, so that the estimation is not only based on the human’s position. Here, it can be determined where the limbs of the human are and whether they are in a vulnerable position. However, the main advantage for acquiring the person’s kinematic state is to differentiate between different implications for possible separation distance violations: Rapid movement of the hand is limited by arm length, but movement of the whole torso is not. Network architectures that are able to capture the persons limbs to generate a topological skeleton (Figure 3D) as in Kendall et al. (2015), Cao et al. (2017), Güler et al. (2018) and Li et al. (2019) are available. Information is typically generated in 2D key-point coordinates, so that an additional distance estimation is required in order o generate world coordinates. Here, deep learning methods can directly determine a 3D position of the human in world coordinates or they can accomplish the construction of volumetric models (Saito et al. (2020), Suo et al. (2021)). Neural Radiance Fields (NeRFs) Mildenhall et al. (2020), Gao et al. (2022) are a recent technique to generate 3D-like representations from a set of 2D images of an object or scene.

The extend of the problem to be solved - detection by simple classification up to 3D reconstruction of limbs—has implications for accuracy, required computing power, remaining uncertainty. Other aspects relate more to hardware issues: Camera resolution and mounting distance, dynamic range, frame rate, and integration time. The dynamic range of standard cameras is still too small to easily cope with shadows, artificial light as well as direct sunlight in the same scene. The resolution is a trade-off between clearly resolving limbs, frame rate, and the input resolution of the network and thus of the available compute resources. To ensure a large viewing area and to avoid occlusion, at least two synchronized cameras should have an overlapping viewing area. In order to avoid occlusion problems and for a simplified distance calculation, we favour ceiling mounted cameras facing downwards. This approach is applicable to both, whole body detection as well as pixel-wise classification. However, it needs to be extended for differentiating limb movements.

**FIGURE 4:**
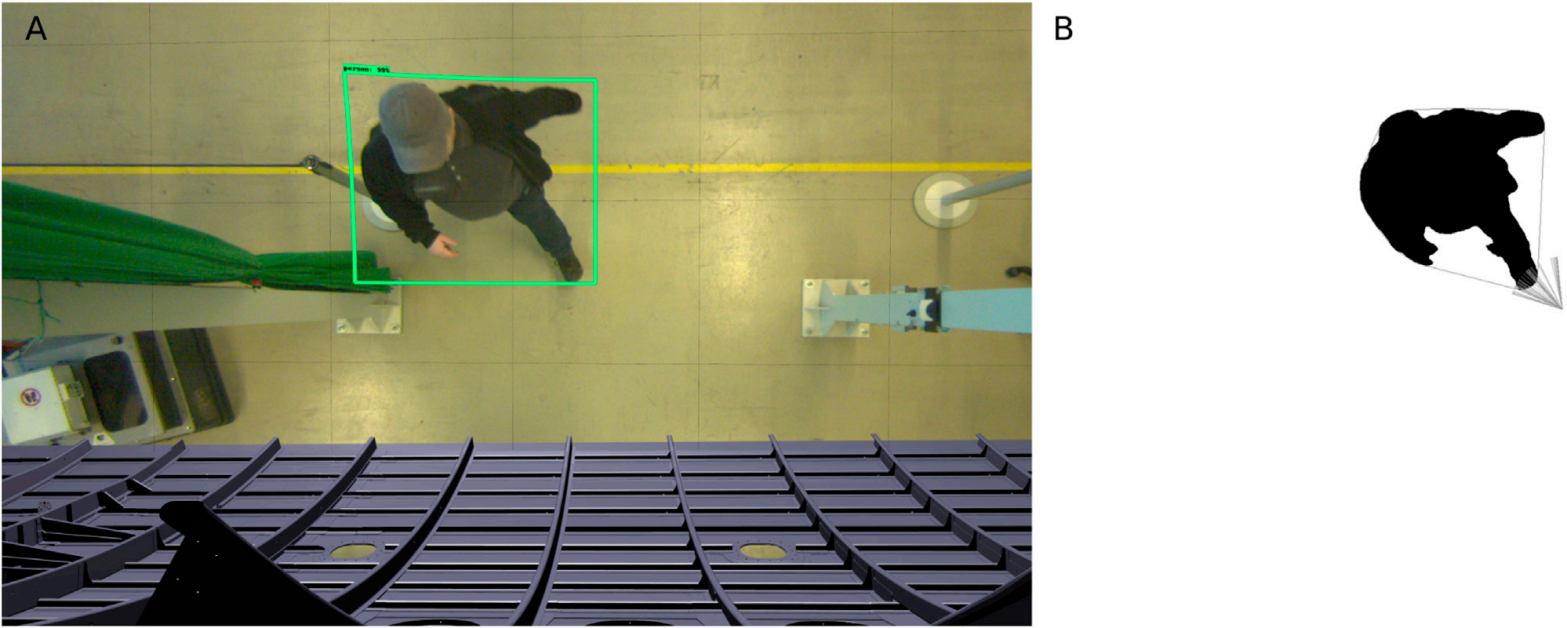
Experiment for detecting a person using top-down perspective and general concept of workspace setup **(A)**; Segmentation result with convex hull and top of 3D-pyramid **(B)**.

We generate the person safety space again in multiple stages (Figure 4). The fist step is selecting an available camera that is not obstructed by either the robot safety space or other structures. We then detect the presence of a human using multiple models running in parallel. Next, the human is then segmented from background (Figure 4A). Next we compute the convex hull of that silhouette. A spatial volume is then constructed by projecting lines from the camera point of view onto the ground floor through the generated hull. In a final step, the resulting pyramid (Figure 4B) is thickened on all sides by the adding sensor uncertainty as safety margin, and finally the hypothetical distance the person could move during the combination of reaction and breaking time. The breaking time is dynamic and is taken from the previous step of calculating the robot safety space. Both volumes of space can then be used together to check whether or not they touch each other. If this is the case, the minimum separation distance would be reached and the stopping of the robot would need to be commenced. When multiple workers are present in the work area, an individual person safety space is generated and checked for intersection with the robot safety space for each individually. We presented results of an experimental setup for detecting humans in an industrial setting using machine learning techniques in Bexten et al. (2020).

## 5 Conclusion and outlook

We have discussed the need for workspace monitoring and detailed separation distance calculation in order to enable intelligent robots in aerospace manufacturing. Application scenarios like the one mentioned in this paper benefit from the capability of human-robot-collaboration at least in the sense of co-existence in shared work spaces. The proposed method for 3D safety space generation which covers all possibilities of braking modes can already be used for analyzing static robot programs at design-time.

Our approach to generating the person safety space is based on generating a 3D representation out of a 2D segmentation of a top-down image in a post-processing step using a silhouette-based algorithm. A future, safety rated implementation of 2D image-based human detection would open up the possibility of deploying our approach. With further development, two interesting improvements are possible. The first one is related to 3D conversion of detected 2D image regions. Here, recent AI-based techniques like NeRFs show promising results when generating 3D representations directly. When considering reliability requirements of safety applications, a combination of multiple AI techniques in a redundant fashion seems to be the most promising approach to future implementations. In these scenarios, the presented algorithm can be applied to a combined 3D representation without any changes. The second improvement is related to differentiating between individual body parts of the recognized persons. This would avoid unnecessarily huge separation distances that are a result of treating every point on the body of a person the same. This would require a future safe implementation of techniques that observe the (approximate) state of a persons kinematic structure like body part recognition, 3D key-point tracking, or similar.

In order to implement the robot-side of the approach in real-world applications, the typical commercial robot controllers used currently need to be replaced. They lack both features and processing power. A robot controller with the capability of pre-planning the trajectory is needed. We also required the robot to safely monitor trajectory execution. The voxel-based computations are expensive in the sense that they require more compute power on the controller in conjunction with high-bandwidth interface to the sensor for volume data exchange.

## Data Availability

The original contributions presented in the study are included in the article/supplementary material, further inquiries can be directed to the corresponding author.
